# Supplementary figures and images for: A six-microRNA signature can better predict overall survival of patients with esophagus adenocarcinoma
Source: PeerJ. 2019 Jul 25;7:e7353. doi: 10.7717/peerj.7353 (PMC6661144; doi:10.7717/peerj.7353)

A

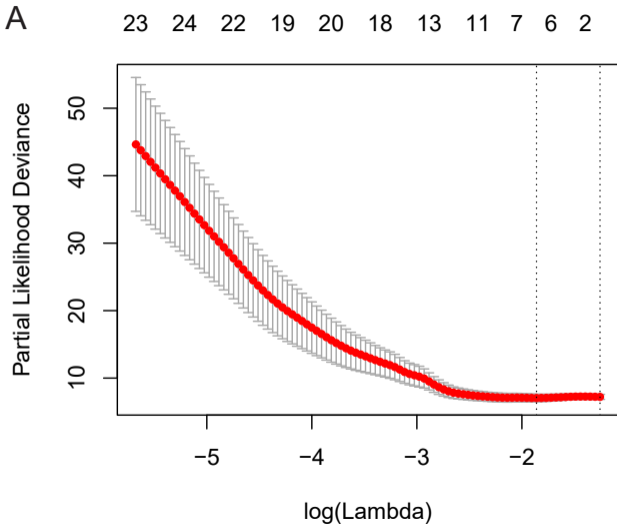

B

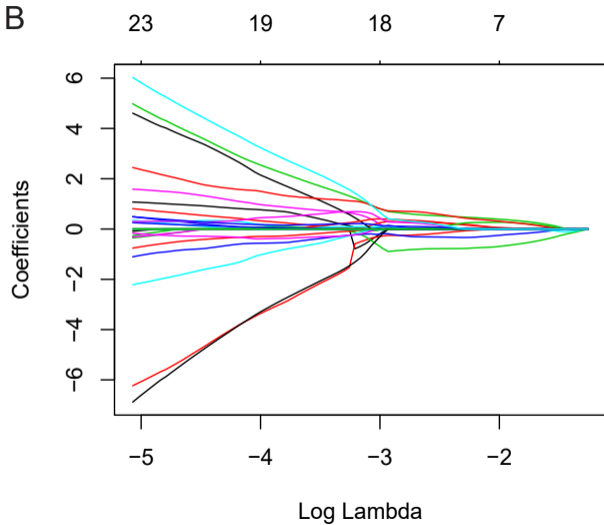

Supplement: Supplemental Information 5 — (A) Lambda selection in the LASSO model used 10-fold cross-validation. Dotted vertical lines on the left and right, respectively, indicate the value with the minimum error and the largest lambda value where the deviance is within one SE of the minimum. (B) LASSO coefficient profiles of the differentially expressed genes associated with the overall survival of EAC. [file peerj-07-7353-s005.pdf]
